# Procalcitonin Testing With Secondary Coinfection in Patients With COVID-19

**DOI:** 10.7759/cureus.28898

**Published:** 2022-09-07

**Authors:** Rashid Nadeem, Hind M Aljaghber, Doaa Elgohary, Aju Rafeeq, Ishma Aijazi, HIba A Khan, Mohammad R Khan, Binu Velappan, Mohanned H Aljanahi, Moatz Galal Mohamed Ali Elzeiny

**Affiliations:** 1 Intensive Care Medicine, Dubai Hospital, Dubai, ARE; 2 Internal Medicine, Dubai Hospital, Dubai, ARE; 3 Intensive Care Unit, Dubai Hospital, Dubai, ARE; 4 Medicine, Dubai Health Authority, Dubai, ARE; 5 Medicine, University of Pikeville, Kentucky College of Medicine, Pikeville, USA; 6 Internal Medicine, Bakhtawar Amin Medical and Dental College, Multan, PAK; 7 Internal Medicine, Dubai Healthcare Authority, Dubai, ARE; 8 Critical Care, Dubai Hospital, Dubai, ARE

**Keywords:** positive cultures, mortality, procalcitonin, secondary infections, covid-19

## Abstract

Background

The coronavirus disease (COVID-19) virus has caused millions of deaths. It is difficult to differentiate between pure viral COVID-19 pneumonia and secondary infection. Clinicians often use procalcitonin (PCT) to decide on empiric antibiotic therapy.

Methodology

We performed a retrospective study of patients admitted with COVID-19 between January 1st, 2020, and June 30th, 2020. Patient demographics, clinical findings, and laboratory findings with a focus on PCT levels were recorded. Coinfection was considered if clinicians ordered a septic workup (urine, blood, and respiratory cultures) or if the physicians started or escalated antimicrobial therapy. PCT levels on the day of culture and daily for the next three days were recorded. Significant PCT change was defined as a decrease in PCT levels of >50% from the initial elevated PCT level.

Results

In total, 143 (59.8%) patients had one secondary infection. These included pulmonary infections (118, 49.4%), blood infections (99, 41.4%), and urine infections (64, 26.8%). Many patients had more than one documented positive culture: respiratory system and blood together in 80 (33.4%) patients, sputum and urine in 55 (23.1%) patients, and urine and blood in 46 (19.2%) patients. Out of the 143 patients with a positive culture, PCT was abnormal on the day of positive culture in 93 (65.5%), while PCT was abnormal in 64 out of 96 on the day of negative culture (66.7%) (p = 0.89). Individual analysis for PCT levels of respiratory cultures showed out of 118 positive sputum cultures, 86 (72%) had abnormal PCT on the day of culture. PCT in positive versus negative cultures was not significantly different, with median PCT (interquartile range, IQR) of 1.66 (6.61) versus 1.03 (2.23) (p = 0.172). For blood cultures, out of 99 positive blood cultures, 73 (73%) had abnormal PCT levels on the day of the culture. PCT in positive versus negative cultures was significantly elevated, with a median of 1.61 (5.97) vs. 0.65 (1.77) (p < 0.001). For urine, out of 64 positive cultures, 41 (64.1%) had abnormal PCT levels on the day of the culture. PCT in positive versus negative cultures was not significantly different, with a median of 0.71 (2.92) vs. 0.93 (4.71) (p = 0.551). To observe the change in PCT after culture, PCT values for the next three days after culture were analyzed. We found that patients with positive cultures had higher PCT levels than those with negative cultures. There was no significant improvement over the following three days. Patients with abnormal PCT on the day of the suspected infection had a longer length of stay in the hospital, with a median (IQR) of 23.9 days (3.16) vs. 16.9 days (2.18) (p = 0.021).

Conclusions

Secondary coinfections in patients with COVID-19 infections are not associated with PCT elevation on the day of suspected secondary infection. However, most patients with bacteremia had a significant elevation of PCT on the day of bacteremia before collection and reporting of positive culture. Patients with abnormal PCT levels on the day of suspected infection had a longer hospital stay than patients with normal PCT levels. Subsequent testing of PCT in patients showed no significant improvement in PCT.

## Introduction

The coronavirus disease 2019 (COVID-19) caused a pandemic starting in December 2019. In a significant number of patients, it leads to adult respiratory distress syndrome requiring intensive care [1]. Bacterial and fungal colonization is frequent in intensive care units (ICUs), and infection with these microorganisms increases mortality [2]. About 14% of patients admitted to ICUs have evidence of coinfection [3]. Most ICU patients also have other risk factors for acquiring secondary fungal or bacterial infections, including immunosuppression, urinary catheters, central lines, endotracheal tubes, and mechanical ventilators [4]. Further, secondary nonviral infections (SNIs) are associated with high morbidity and mortality [5].

Clinical assessment to suspect secondary coinfection is difficult. Patients may have a fever, increase in white blood cell (WBC) count, purulent secretions, and development of new infiltrates. The progression of COVID-19 infection also has the same signs. Septic shock and high lactate levels in patients suggest secondary infection. COVID-19 patients may develop septic shock from viral infection alone. These patients are treated with sedation and neuromuscular paralysis which may also cause hypotension. Additionally, liver dysfunction may cause high lactate levels in these patients. New infiltrates on the chest radiograph may suggest a secondary infection. Atelectasis and infiltrates are common with COVID-19 pulmonary infection alone. The use of analgesics and antipyretics often masks new fever and makes it difficult to detect a new infection. The high patient load during the pandemic did not allow physicians to visit individual patients multiple times. Therefore, reliance on laboratory testing to detect these coinfections increased. We observed a high number of procalcitonin (PCT) tests performed sometimes even in daily morning laboratory testing. Physicians believed that abnormal PCT levels may alert them to look for secondary infection. We aim to investigate if PCT levels are associated with secondary coinfections. We also calculate the impact of PCT levels on the clinical outcome of the length of hospital stay.

## Materials and methods

We performed a retrospective review of all patients admitted to the ICU of Dubai Hospital with COVID-19 between January 1st, 2020, to June 30th, 2020. Dubai Scientific Research Committee approved the study after a formal review (approval number: DSREC-07/2020_10). Exclusion criteria were age <18 years and non-COVID-19 patients. We collected the following data: age, body mass index, medical conditions, immune status, and APACHE-2 scores. The following laboratory data were also recorded: oxygenation, ventilation parameters, ventilator settings, and laboratory values of complete blood count, renal function, C-reactive protein, D-dimer, and ferritin. Coinfection was considered if the clinician ordered a septic workup (urine, blood, and respiratory cultures) or if the physician started or escalated antimicrobial therapy. PCT levels on the day of the culture and daily for the next three days were also recorded (if available). Significant PCT change was defined as a decrease in PCT levels of >50% from the initial elevated PCT value.

We also recorded data regarding the treatment provided, which included antiviral medications, renal replacement therapy, extracorporeal membrane oxygenation, and pronation therapy. We recorded hospital length of stay as an outcome measure. Culture studies were also recorded to document secondary pneumonia, bacteremia, catheter infections, and urinary tract infections. We also collected data about *Clostridium difficile* colitis. We did not collect data for secondary viral infections as it is not a routine practice in ICUs. The use of sedation, neuromuscular blocking agents, and vasopressors was also recorded.

We compared the data (PCT levels) between patients with secondary infections and those without secondary infections by statistical comparison of mean and standard deviation (SD) if the data distribution was normal. For categorical variables, we performed the Fisher exact test. For skewed distribution, median and interquartile range (IQR) were compared for continuous variables, and numbers and percentages were reported for categorical variables. All tests were two-sided, and p-values of <0.05 were chosen to indicate statistical significance. SPSS software version 22.2 (IBM Corp., Armonk, NY, USA) was used for statistical analysis.

## Results

Patient characteristics are provided in Table 1 for categorical variables and in Table 2 for continuous variables.

**Table 1:** Patient characteristics (categorical variables). *Chi-square to compare categorical variables. CRRT: continuous renal replacement therapy; ECMO: extracorporeal membrane oxygenation

	All patients	Secondary infection	No secondary infection	
Clinical features	Number = 237	Number = 119	N = 118	P-value*
Male (%)	208 (87.8)	108 (90.8)	100 (84.7)	0.158
Cough	190 (80.5)	99 (83.2)	91 (77.8)	0.294
Fever	216 (91.1)	111 (93.3)	105 (89)	0.245
Dyspnea	191 (80.6)	99 (83.9)	92 (77.3)	0.200
Gastric symptoms	29 (12.9)	14 (12.6)	15 (13.2)	0.903
Diabetes	103 (43.3)	48 (40.3)	55 (46.2)	0360
Hypertension	59 (25)	28 (23.5)	31 (26.5)	0.599
Coronary artery disease	16 (6.8)	7 (5.9)	9 (7.6)	0.617
Renal disease	25 (10.8)	15 (13)	10 (8.5)	0.269
Outpatient dialysis	16 (6.7)	13 (10.9)	3 (2.5)	0.010
Immunodeficiency	10 (4.3)	5 (4.3)	5 (4.3)	0.100
Clinical variables				
Inpatient fever	205 (86.5)	108 (90.8)	97 (82.2)	0.054
Tachycardia	187 (78.6)	103 (86.6)	84 (70.6)	0.003
Hypotension	120 (50.4)	76 (63.9)	44 (37)	0.001
Hypoxia	204 (85.7)	109 (91.6)	95 (79.8)	0.010
Mechanical vent	203 (85.3)	111 (93.3)	92 (76.7)	0.001
Vasopressers	187 (78.9)	108 (90.8)	79 (66.9)	0.001
CRRT	73 (30.7)	52 (43.7)	21 (17.6)	0.001
Treatment				
Chloroquine	209 (88.2)	101 (85.6)	108 (90.8)	0.218
Lopinavir/Ritonavir	86 (36.4)	50 (42.7)	36 (30.3)	0.046
Favipiravir	190 (80.2)	97 (82.2)	93 (78.2)	0.434
Steroids	188 (79.3)	106 (89.8)	82 (68.9)	0.001
Tocilizumab	37 (15.7)	24 (20.5)	13 (10.9)	0.043
Tracheostomy	31 (13)	26 (21.8)	5 (4.2)	0.001
ECMO	13 (5.5)	11 (9.2)	2 (1.7)	0.010
Sedatives	211 (88.7)	118 (99.2)	93 (78.2)	0.001
Narcotics	181 (76.7)	100 (76.7)	81 (69.2)	0.007
Neuromuscular blocking agents	202 (84.9)	113 (84.9)	89 (74.8)	0.001
Anticoagulation	229 (97)	117 (99.2)	112 (94.9)	0.055
GI prophylaxis (proton pump inhibitors)	224 (96.6)	115 (99.1)	109 (94)	0.031
Mortality	119 (50.4)	67 (57.3)	52 (43.7)	0.037

**Table 2:** Sample characteristics (continuous variables). BMI: body mass index; WBC: white blood cell; CRP: C-reactive protein; CPK: creatine phosphokinase; ABG: arterial blood gas; LOS: length of stay; ICU: intensive care unit

Continuous variables	Total, N = 237	Secondary infection, N = 119	No secondary infection, N = 118	P-value
	Median (IQR)	Median (IQR)	Median (IQR)	
Age (years)	49 (42–57)	50 (44–57)	47.0 (41.0–56)	0.105
BMI (kg/m^2^)	27.5 (25–31)	27.5 (25–31.3)	27.6 (25.0–31)	0.939
WBC (10^3^/µL)	7.9 (6–10.8)	7.7 (6–10.7)	8.0 (6.1–11.6)	0.503
Ferritin (ng/mL)	1,293 (577–1945)	1,270 (706.3–2,131)	1,319.5 (470.4–1,845)	0.080
D-dimer (ng/mL)	1.17 (0.57–3.73)	1.21 (0.61–4.05)	1.1 (0.5–3.3)	0.290
Procalcitonin (ng/mL)	0.34 (0.13–1.09)	0.34 (0.15–1.16)	0.3 (0.1–1)	0.473
CRP (mg/L)	129 (75–215)	122 (72–218)	135.0 (77.0–215.8)	0.594
Creatinine (mg/dL)	0.9 (0.8–1.2)	1 (0.8–1.3)	0.9 (0.7–1.2)	0.016
CPK (U/L)	226 (96–653)	29 (114.5–887)	175 (79.3–482.8)	0.015
ABG pH	7.38 (7.28–7.43)	7.39 (7.27–7.44)	7.4 (7.3–7.4)	0.686
pCO_2_ (Torr)	37.3 (31.3–47.3)	37.7 (30.8–47.3)	35.4 (31.9–47.5)	0.992
pO_2_ (Torr)	63 (47–90)	66.9 (50.8–86.1)	59.8 (45.9–93.6)	0.260
Lactate (mmol/L)	1.7 (1.3–2.5)	1.7 (1.2–2.7)	1.7 (1.3–2.4)	0.976
Bicarbonate (mEq/L)	21.6 (18.9–24)	22 (19.2–24.3)	21.2 (18.9–24)	0.458
Magnesium (mg/dL)	2.04 (1.9–2.2)	2.03 (1.9–2.25)	2.1 (1.9–2.3)	0.338
Platelets (10^3^/µL)	203 (155–263)	185.5 (146.75–255.7)	222.0 (172.0–285.5)	0.014
Days on mechanical ventilation	11 (4.2–20.7)	19 (11–27.2)	5.0 (2.0–10)	<0.001
LOS ICU (days)	14 (5–23)	22 (15–33.2)	7.0 (2.0–11)	<0.001
Hospital LOS (days)	19 (9–32)	28 (18–45.5)	11.5 (6.0–19)	<0.001
APACHE 2 scores	16 (13–21)	16 (12.7–20.2)	17.0 (13.0–22)	0.582

In this study, we defined positive cultures as cases and negative cultures as controls. We compared PCT levels of culture-positive (cases) to culture-negative (controls) for sputum, blood, and urine. A significantly high level of PCT for positive cultures versus negative cultures was noted for sputum, blood, and urine on the day of the cultures and the subsequent three days (Table 3).

**Table 3:** Comparison of procalcitonin levels between culture positives and culture negatives for three consecutive days. *Median (interquartile range). PCT: procalcitonin

	Positive sputum culture	Negative sputum culture	P-value	Positive blood culture	Negative blood culture	P-value	Positive urine culture	Negative urine culture	P-value
PCT on the day of the culture	1.65 (6.46)*	0 (0.1)	0.01	1.57 (5.91)	0.29 (1.11)	0.01	0.95 (2.94)	0.52 (2.28)	0.01
PCT one day after the culture	1.48 (8.18)	0 (0)	0.01	1.87 (11.3)	0(0.47)	0.01	0.87 (3.40)	0.17 (2.47)	0.01
PCT two days after the culture	1.01 (5.44)	0 (0)	0.01	1.2 (12.07)	0 (0.57)	0.01	0.94 (3.97)	0 (2.03)	0.01
PCT three days after the culture	0.96 (4.52)	0 (0)	0.01	0.88 (6.33)	0 (0.78)	0.01	0.86 (5.68)	0 (1.3)	0.01

We found high rates of coinfection. In total, 143 (59.8%) patients had one secondary infection (respiratory, blood, or urine). These included sputum (118, 49.4%), blood (99, 41.4%), and urine (64, 26.8%). Many patients had two or more anatomic sites with positive cultures at the same time. Sputum and blood in 80 (33.4%) patients, sputum and urine in 55 (23.1%) patients, and urine and blood in 46 (19.2%) patients.

PCT was not significantly different in patients with positive cultures (cases) versus negative cultures (controls) (93 (65.5%) vs. 64 (66.7%); p = 0.89).

We performed individual analyses for respiratory, blood, and urine cultures. First, we analyzed if the data were normally distributed. We found that data were not normally distributed. Therefore, the median with IQR of PCT of positive cultures versus negative cultures was compared using the Mann-Whitney U test. For respiratory cultures, no significant difference was found, with a median (IQR) of 1.66 (6.61) vs. 1.03 (2.23) (p = 0.172). For bacteremia, PCT was significantly higher in culture-positive patients compared to culture-negative patients (1.61 (5.97) vs. 0.65 (1.77); p < 0.001). For urine, no significant difference was found (0.71 (2.92) vs. 0.93 (4.71); p = 0.551).

We recorded the change in PCT for daily follow-up three days after culture. If the PCT was decreased by >50%, it was considered an improvement. There was no significant improvement in PCT levels during the subsequent three days.

For the outcome of the length of hospital stay, we assessed if the length of hospital stay data were normally distributed. Because the data were not normally distributed, the length of hospital stay was converted into a log value. We observed the interaction between abnormal PCT levels on the days of the culture and log of length of hospital stay and found that patients with abnormal PCT levels had a higher length of hospital stay than those who had normal PCT, with a median (IQR) of 23.9 days (3.16) vs. 16.9 days (2.18) (p = 0.021).

## Discussion

We assessed the relationship between PCT and secondary infections and compared the PCT levels of patients with positive cultures versus those with negative cultures. In our sample, the coinfection rate was high, with >50% of patients having at least one infection. Previous studies have reported coinfection rates ranging from 10% to 45% [6-8]. Our study sample had positive blood cultures in 84 (35.4%). We noted positive respiratory cultures in 72 (30.3%) patients. Urinary cultures were positive in 36 (15.1%) patients. Studies on COVID-19 patients have reported the rate of secondary fungal infections as 6.3% (0.9-33.3%) [9].

We found that patients with positive cultures did not have higher PCT levels than those with negative cultures. Therefore, frequent PCT testing did not help alert physicians toward coinfection except in cases of bacteremia. The following reasons may explain this. As most daily morning labs are drawn between 0400 and 0500 and patients may acquire infection later in the day (next 24 hours), it may not be elevated. Many patients have additional reasons for PCT elevation other than infection, for example, renal failure and cardiovascular collapse requiring cardiopulmonary resuscitation [10]. Another reason is that fungal infections are not associated with PCT elevation.

Comparing PCT levels of positive blood cultures with PCT levels of negative blood cultures, we found that PCT levels were significantly high in positive blood cultures (bacteremia). This suggests that bacteremia may be a more invasive and serious form of infection; therefore, PCT is most likely elevated early on. Atallah et al. also showed that PCT helps detect bacteremia [11]. Because we did not divide our patients into septic or non-septic patients with multiorgan failure, it is possible that there may be a robust role of PCT in septic patients which we may have missed.

PCT has been used as a predictive marker in bacterial infections in emergency departments [12]. Another study documented that PCT may be an indicator of disease severity and may contribute to determining the severity of patients with COVID-19 [13]. A recent study suggested that PCT (>0.5 μg/L) could be an important prognostic indicator for hyperinflammation and the cytokine storm typically seen in severe COVID-19 [14]. It can be extremely difficult to distinguish a cytokine storm in a viral infection from a cytokine storm in bacterial sepsis.

We found no significant improvement in PCT levels on daily follow-up for three days. We understand that three days may not be enough time to observe a decreasing trend in PCT. We chose to record three days only as most clinicians review PCT to observe treatment effects for three days. If there is no significant decrease they consider the antimicrobial treatment to be inadequate. A meta-analysis involving non-COVID-19 patients showed that PCT guidance can safely reduce antibiotic usage when used to discontinue antibiotic therapy in adult ICU patients [15]. A multicenter trial using a PCT-based algorithm showed a reduction in antibiotics and antibiotic discontinuation was encouraged if PCT had decreased >80% from baseline or PCT absolute value of <0.5 ng/mL. The mean antibiotic duration declined significantly from 13 days to 9.5 days for the PCT-guided group. The study also showed mechanical ventilation-free days, 28-day mortality, and length of hospital stay remained unchanged [16]. Studies of COVID-19 patients have documented stopping antibiotics in patients with normal PCT without an increase in mortality [17]. Another study showed that normal PCT on admission correlates with shorter antimicrobial courses and early stopping of therapy as well as predicts a lower frequency of ICU admission [18]. The difference in our results and others could be for many reasons. The focus of these studies was PCT level on admission in relatively stable non-ICU patients. We studied severe COVID-19 acute respiratory distress syndrome patients admitted to the ICU. Differences in the patient population and designs of studies could also produce different results.

We observed that patients with high PCT levels have a longer hospital stay. Jackson et al. [19] studied PCT levels on admission to the hospital and found no difference in the length of hospital stay comparing low (<0.25 ng/mL) versus high PCT (>0.5 ng/mL). We previously reported no impact of a single value of PCT level drawn on admission to the length of hospital stay in our cohort of 391 patients [20].

Our study had some limitations. It is a retrospective study with a small sample size. Clinical care was provided by the physician taking care of the patient, not under any research protocol. Because we analyzed culture-proven infections only, we may have missed culture-negative infections. We do not perform routine bronchoscopies and bronchoalveolar lavage in our ICU. Therefore, we might have missed fungal infections such as *Aspergillus*. Others have documented *Aspergillus *infections as they perform bronchoalveolar lavage routinely [21,22]. Finally, the study design did not include non-COVID-19 patients, therefore our observations are only limited to COVID-19 patients.

## Conclusions

Secondary coinfections in patients with COVID-19 infections are not associated with PCT elevation. However, most patients with bacteremia had a significant PCT elevation on the day of bacteremia. Patients with abnormal PCT levels on the day of suspected infection and cultures had a longer hospital stay than those with normal PCT levels. Subsequent levels of PCT three days following infection in patients with infection did not show any significant improvement.
